# Limits to the thermal tolerance of corals adapted to a highly fluctuating, naturally extreme temperature environment

**DOI:** 10.1038/srep17639

**Published:** 2015-12-02

**Authors:** Verena Schoepf, Michael Stat, James L. Falter, Malcolm T. McCulloch

**Affiliations:** 1 School of Earth and Environment and ARC Centre of Excellence for Coral Reef Studies, The University of Western Australia, Crawley, WA, Australia; 2 UWA Oceans Institute, The University of Western Australia, Crawley, WA, Australia; 3 The Western Australian Marine Science Institution, Perth, WA, Australia; 4 Trace and Environmental DNA (TrEnD) Laboratory, Department of Environment and Agriculture, Curtin University, Perth, WA, Australia

## Abstract

Naturally extreme temperature environments can provide important insights into the processes underlying coral thermal tolerance. We determined the bleaching resistance of *Acropora aspera* and *Dipsastraea* sp. from both intertidal and subtidal environments of the naturally extreme Kimberley region in northwest Australia. Here tides of up to 10 m can cause aerial exposure of corals and temperatures as high as 37 °C that fluctuate daily by up to 7 °C. Control corals were maintained at ambient nearshore temperatures which varied diurnally by 4-5 °C, while treatment corals were exposed to similar diurnal variations and heat stress corresponding to ~20 degree heating days. All corals hosted *Symbiodinium* clade C independent of treatment or origin. Detailed physiological measurements showed that these corals were nevertheless highly sensitive to daily average temperatures exceeding their maximum monthly mean of ~31 °C by 1 °C for only a few days. Generally, *Acropora* was much more susceptible to bleaching than *Dipsastraea* and experienced up to 75% mortality, whereas all *Dipsastraea* survived. Furthermore, subtidal corals, which originated from a more thermally stable environment compared to intertidal corals, were more susceptible to bleaching. This demonstrates that while highly fluctuating temperatures enhance coral resilience to thermal stress, they do not provide immunity to extreme heat stress events.

Coral reefs are in serious decline worldwide^1^ and increasingly suffer from episodes of thermally induced stress or coral bleaching, which lead to the breakdown of the vital endosymbiosis with dinoflagellates in the genus *Symbiodinium* spp.^2^,^3^. Corals typically obtain the majority of their metabolic requirements from photosynthetic carbon translocated from their endosymbionts^4^. Thus, the loss of these symbionts via bleaching significantly reduces their ability to meet key metabolic needs and can ultimately lead to death if continued for a prolonged period of time. As surface ocean temperatures have already increased on average by 0.6 °C since preindustrial times and are projected to increase by at least another 2 °C under a business as usual scenario by the year 2100^5^, coral bleaching events are expected to increase in frequency and intensity over the coming decades^6^,^7^. This raises the question of whether corals are capable of acclimatising and/or adapting to not only rising ocean temperatures but also more frequent extreme thermal events, and if so, whether these processes will be fast enough to keep pace with the rapid rates of ocean warming that are currently occurring.

The majority of coral reefs occur in tropical latitudes between 22 °S and 22 °N and thus only experience relatively limited seasonal changes in water temperatures (4-5 °C) and average maximum temperatures of ~30 °C^8^. However, coral reefs also exist in much more extreme temperature environments such as the Persian/Arabian Gulf (referred to as “the Gulf” hereafter) where the seasonal temperature range can be >20 °C (14–36 °C) and daily mean summer temperatures can reach 34-35 °C for several months^9^. The existence of such communities demonstrates that corals can adapt to a large range of temperatures. In addition to adaptation involving genetic change, thermal tolerance can also be influenced by acclimatization, which involves non-heritable adjustments in response to an organism’s environment that occur within its lifetime. Consequently, upper limits of thermal tolerance vary significantly over both large and small spatial scales^10^,^11^.

Furthermore, corals living in thermally more variable environments such as those found in back reefs or on reef flats are often found to be more resistant to temperature stress and bleaching compared to corals from thermally more stable environments such as the fore reef^12^,^13^,^14^,^15^, although this is not always the case^16^. Therefore, thermally variable environments seem to enhance coral thermal tolerance beyond that determined by maximum summer temperatures and can therefore promote an increased resistance to climate change. This may partly explain why hindcast predictions of bleaching events based on historical temperature variability rather than climatological maxima showed greater predictive power^17^.

The degree to which coral can resist bleaching is further influenced by their particular physiological aspects such as morphology, symbiont genotype, tissue characteristics and capacity for particle feeding. For example, branching corals are typically more susceptible to bleaching than massive corals^18^,^19^, and corals hosting *Symbiodinium* clade D are often, though not always, more tolerant of thermal stress than corals hosting other symbiont types^20^,^21^. Thick tissues and high levels of stored energy reserves also promote further resistance to bleaching^22^,^23^, while the capacity to increase heterotrophic feeding during bleaching can help some corals avoid resource limitation and starvation^24^,^25^.

Corals living in naturally extreme temperature environments can provide important insight into the mechanisms underlying coral resistance to thermal stress. However, our present knowledge of these mechanisms has come mainly from a few sites (e.g., the southern Gulf^11^ and the back reef pools of American Samoa^15^). Given the importance of understanding how corals will ultimately respond to current rates of ocean warming, it is therefore critical to study the growth of reef-building corals in as wide a range of naturally extreme temperature environments as possible. The little-known Kimberley region in northwest Australia is a naturally extreme environment that supports unusual and highly diverse coral reefs^26^,^27^,^28^,^29^, yet remains poorly studied due to its remote location and difficulty of access. This region is characterized by the largest tropical tides in the world (up to 10 m during spring tides), strong currents and turbid waters^26^,^28^,^29^. Corals living in the subtidal Kimberley are thus adapted to naturally high mean water temperatures which exceed 30 °C for five months of the year^26^,^27^ (Fig. 1). Due to the extreme tides, intertidal corals often experience significant short-term temperature fluctuations of up to 7 °C daily as well as aerial exposure for several hours^26^,^27^,^28^,^29^. Yet these intertidal coral show no obvious signs of stress and even calcify at rates that are comparable to congeners from more typical tropical reef settings^27^. Thus although the Kimberley region is comparable to other naturally extreme temperature environments such as the back reef pools of American Samoa^30^, though not quite as warm as the Gulf^9^, it differs in being a much more dynamic and variable environment. In particular, the large tidal range and frequent aerial exposure of intertidal corals provides a unique set of environmental conditions to study the scope and limits for coral thermal tolerance and adaptation in the face of climate change.

The existence of coral reefs in such naturally extreme and variable temperature environments is encouraging in view of global warming, but it remains unclear whether their increased tolerance to highly variable temperatures also implies an increased tolerance to the long-term shifts in ocean temperature expected to occur over the 21^st^ century (≥2 °C). Therefore, the goal of this study was to experimentally assess the tolerance to variable and elevated water temperatures of two common Kimberley corals (branching *Acropora aspera* and massive *Dipsastraea* sp. (formerly *Favia*^31^)) from both intertidal and subtidal environments (Fig. 1). We hypothesised that (1) Kimberley corals have higher bleaching thresholds than expected based on local mean summer temperatures due to the naturally extreme thermal environment, (2) corals from the intertidal environment are more resistant to thermal stress than subtidal corals due to the more pronounced daily temperature fluctuations that they experience *in situ* (Fig. 1, see Methods), and (3) *Dipsastraea* corals are more resistant than *Acropora* independent of their original environment. To test these hypotheses, corals were subjected to either ambient control temperatures, ambient +2 °C or ambient +3 °C (Fig. 2) for 11 days in outdoor flow-through seawater tanks during which we followed changes in key metrics of both symbiont and coral physiology (see Methods).

## Results

For 1 week prior to the start of the experiment, all corals were allowed to acclimate to ambient treatment conditions, including a daily temperature variation of 4-5 °C (see Methods). All corals appeared visibly healthy at the beginning of the experiment, and all ambient control corals appeared to remain healthy throughout the experiment (see also Figs 3 and 4A,B). Average day and night temperature, degree heating days, heating rate, pH_T_, pCO_2_, total alkalinity, saturation state and nutrient concentrations for each of the six tanks are summarized in Table 1. None of these parameters differed significantly between intertidal and subtidal tanks subject to the same temperature treatment (Table S1). Thus, any observed differences in the response of intertidal versus subtidal corals within each temperature treatment can be attributed to differences in their *in situ* habitat and are independent of tank effects.

Temperature profiles for each temperature treatment, including the gradual increase of 0.6 °C per day until target temperatures were reached in the two stress treatments, are shown in Fig. 2. From day 6 to 9, unusual weather conditions associated with storms, high cloud cover and strong winds resulted in cooler water temperatures, particularly in the ambient +3 °C treatment where the heater struggled to maintain the high temperature under these conditions (Fig. 2). Therefore, this treatment was not consistently higher than the ambient +2 °C treatment for the entire experiment resulting in similar average day and night temperatures, degree heating days and heating rate for the two treatments (Table 1). However, temperatures in the +3 °C treatment were higher than in the +2 °C treatment on days 1, 4, 5, 10 and 11 and during night 4 (Fig. 2); thus, resulting in an overall more variable and stressful treatment, the effect of which became evident in the physiological data (e.g., chlorophyll *a* fluorescence, Figs 3 and 4, see below).

### Photophysiology and Mortality

#### Acropora

Active chlorophyll *a* fluorescence is generally the preferred method for detecting the initial onset of heat-stress induced coral bleaching^32^. Over the duration of the experiment, maximum photosynthetic quantum yield (Fv/Fm) of *Acropora* corals decreased significantly with time in both heat stress treatments (+2 °C and +3 °C, Table S2), while remaining high and relatively constant in the ambient controls regardless of whether the corals were from the intertidal or subtidal environment (Fig. 3A,B). Furthermore, Fv/Fm of heat-stressed corals from the +3 °C treatment declined sooner and reached significantly lower values compared to corals in the +2 °C treatment for much of the experiment. This trend was also reflected in the excitation pressure over photosystem II (Qm) (Fig. 3C,D, Table S2), which is the ratio of the effective quantum yield at midday relative to the maximum quantum yield (see Methods). However, the gap between the greater decline in Fv/Fm as well as the greater increase in Qm of corals from the +3 °C versus the +2 °C treatment narrowed during the last days of the experiment (Fig. 3A,B).

Despite these overall similar trends, subtidal *Acropora* showed much greater declines in Fv/Fm than intertidal *Acropora* corals, with Fv/Fm being 51–57% lower in heat-stressed subtidal *Acropora* relative to controls after 11 days of heat stress and only 32-33% lower in heat-stressed intertidal *Acropora* (Fig. 3A,B, Table S2). This increased susceptibility to heat stress in subtidal versus intertidal heat-stressed *Acropora* was also evident in much higher values of Qm throughout much of the experiment (Fig. 3C,D, Table S2).

Within 5-6 days of heat stress, many *Acropora* corals became highly susceptible to rapid tissue necrosis (RTN), which resulted in tissue sloughing and death within 24–48 hours (Fig. 3E,F). Similar to trends in photophysiology, mortality occurred both earlier and at a higher rate in subtidal versus intertidal corals (Fig. 3E,F). By the end of the experiment, 75% of all heat-stressed subtidal *Acropora* had died (+2 °C and +3 °C treatments, Fig. 2F), whereas only 50–58% of all heat-stressed intertidal *Acropora* had died (Fig. 2E). Importantly, neither subtidal nor intertidal *Acropora* in the ambient control treatment developed RTN or died (Fig. 3E,F).

#### Dipsastraea

Similarly to *Acropora*, all heat-stressed *Dipsastraea* corals from both environments showed significant declines in Fv/Fm over the course of the experiment, while control corals maintained high and relatively stable values (Fig. 4A,B, Table S3). However, intertidal *Dipsastraea* showed similar declines in Fv/Fm in both the +3 °C and the +2 °C treatment (Fig. 4A), whereas subtidal *Dipsastraea* from the +3 °C treatment had significantly lower Fv/Fm values than corals in the +2 °C treatment from day 4 onward (Fig. 4B). Furthermore, subtidal *Dipsastraea* overall experienced greater declines in Fv/Fm than intertidal corals, with 37–48% lower values relative to controls observed in subtidal corals at the end of the experiment compared to only 30–32% lower values in intertidal corals (Fig. 4A,B).

Levels of Qm in heat-stressed *Dipsastraea* corals were generally lower than in *Acropora* regardless of their original environment (Fig. 4C,D). Further, intertidal heat-stressed *Dipsastraea* generally experienced similar Qm values as the controls for the majority of the experiment (Fig. 4C, Table S3). In contrast, subtidal *Dipsastraea* in the ambient +2 °C treatment had significantly lower Qm values than the controls on 5 out of the 11 days (days 4, 6, 8, 9, and 10; Fig. 4D). Subtidal *Dipsastraea* in the ambient +3 °C treatment had significantly higher Qm than the controls on day 3, but otherwise did not differ significantly from the controls (Fig. 4D).

In stark contrast to *Acropora*, none of the *Dipsastraea* corals from either the elevated or ambient temperature treatments developed RTN or died despite being maintained in the same tanks as the *Acropora* corals (Fig. 4E,F).

### Endosymbiont Type

A total of 79 *Symbiodinium* chloroplast 23S rDNA sequences were recovered from the coral fragments used in the experiment. All sequences belonged to clade C *Symbiodinium* and were thus independent of species, treatment or environment. There were two unique clade C haplotypes: 76 sequences were identical to Cp1 (accession number FJ461478^33^), and three sequences represented a novel haplotype Cp20 (KT223627) that is a single base pair different to Cp1. The three coral fragments with *Symbiodinium* Cp20 all originated from the same parent colony (subtidal *Acropora* #8).

### Chlorophyll *a*, Symbiont Density and Tissue Biomass

#### Acropora

Area-normalized chlorophyll *a* concentrations were significantly lower in heat-stressed *Acropora* corals relative to ambient controls (Fig. 5A, Table S4), with this effect being more pronounced in colonies from the subtidal versus intertidal environment (−73% and −91% versus −51% and −60% for the +2 °C and +3 °C treatments, respectively; Table S4). The effect of heat stress on chlorophyll *a* concentrations was much less pronounced when normalizing per symbiont cell rather than per surface area for both the subtidal and intertidal colonies: heat-stressed intertidal *Acropora* in the ambient +2 and +3 °C treatments had only 14% and 21% lower concentrations than the controls, respectively, but in heat-stressed subtidal *Acropora* they were 30% higher and 38% lower, respectively (Fig. 5B, Table S4). This more damped response in chlorophyll *a* per cell versus per area was due to significant declines in symbiont densities within heat-stressed corals (Fig. 5C, Table S4), the effect of which was again more pronounced in colonies from the subtidal versus intertidal environment (−79% and −86% versus −58% and −65% for the +2 °C and +3 °C treatments, respectively). Finally, tissue biomass was not significantly influenced by either temperature or environment (Fig. 5D, Table S4); however, heat-stressed intertidal *Acropora* corals tended to have a 23–26% lower biomass than the controls (Fig. 5D).

#### Dipsastraea

Heat-stressed corals had significantly lower area-normalized chlorophyll *a* concentrations than the controls, and subtidal corals generally had lower concentrations than intertidal corals (Table S4, Fig. 5E). Specifically, heat-stressed intertidal *Dipsastraea* in the ambient +2 and +3 °C treatments had 49% and 50% lower concentrations than the controls, respectively, whereas concentrations were 58% and 65% lower in subtidal *Dipsastraea*, respectively (Fig. 5E). Similar to *Acropora*, the effect of heat stress on *Dipsastraea* chlorophyll *a* concentrations was much less pronounced when normalizing per symbiont cell rather than per surface area for coral from both environments, and corals in the ambient +2 °C treatment had the lowest concentrations (Fig. 5F, Table S4). This was generally due to significant declines in symbiont density in heat-stressed *Dipsastraea* corals, with more pronounced declines in heat-stressed subtidal corals (−43% and −58% versus −34% and −38% in the +2 °C and +3 °C treatments, respectively; Fig. 5G, Table S4). Finally, tissue biomass was significantly higher (+19%) in intertidal than subtidal *Dipsastraea* corals, however no temperature effect was observed (Table S4, Fig. 5H).

Results for chlorophyll *c*_*2*_ per area and per cell were similar to those for chlorophyll *a* in both corals (Fig. S1, Table S5).

## Discussion

### How resistant are Kimberley corals to heat stress and bleaching?

**Figure 1:**
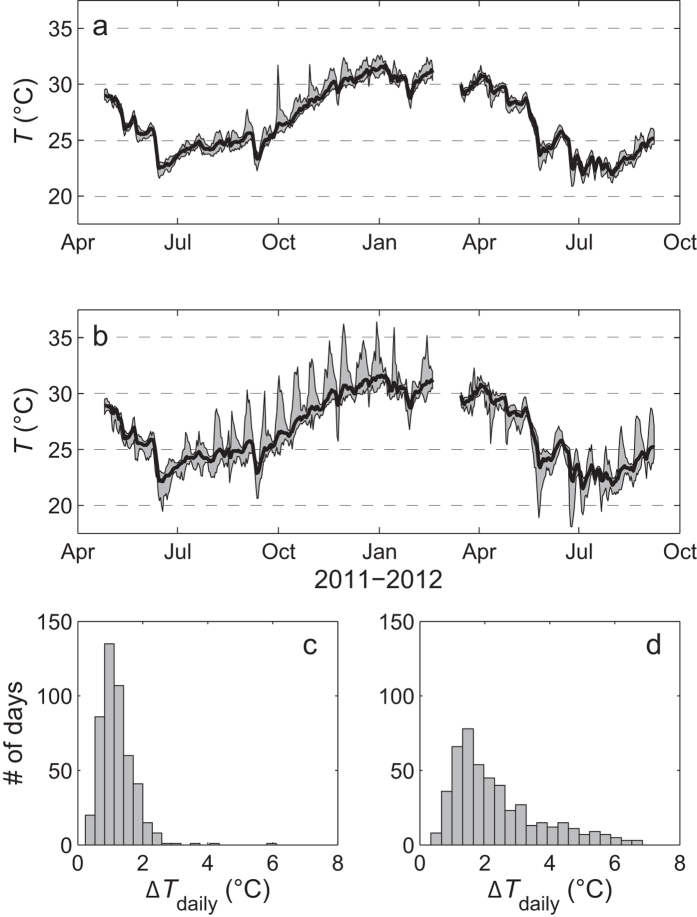
Temperature data for the (a) subtidal and (b) intertidal environment from 2011-12^27^. Histograms show the number of days with a certain daily temperature range (ΔT_daily_) in the (**c**) subtidal and (**d**) intertidal environment for the same time period. In panels (**a**,**b**) the bold black line shows the mean daily temperature, while the hourly max. and hourly min. temperature for each day is shown as a grey envelope around the daily mean.

The present study is the first to examine the thermal tolerance of corals growing in the remote Kimberley region of north-western Australia. Despite the fact that corals growing in this region experience large daily temperature variability (up to 7 °C) and temperature extremes of up to 37 °C^27^ (Fig. 1), branching *Acropora* and massive *Dipsastraea* corals were highly susceptible to coral bleaching when exposed to heat stress corresponding to ~20 degree heating days. For *Acropora*, this further resulted in up to 75% mortality due to rapid tissue necrosis and tissue sloughing, potentially due to an increased sensitivity to the pathogen *Vibrio* spp.^34^.

**Figure 2:**
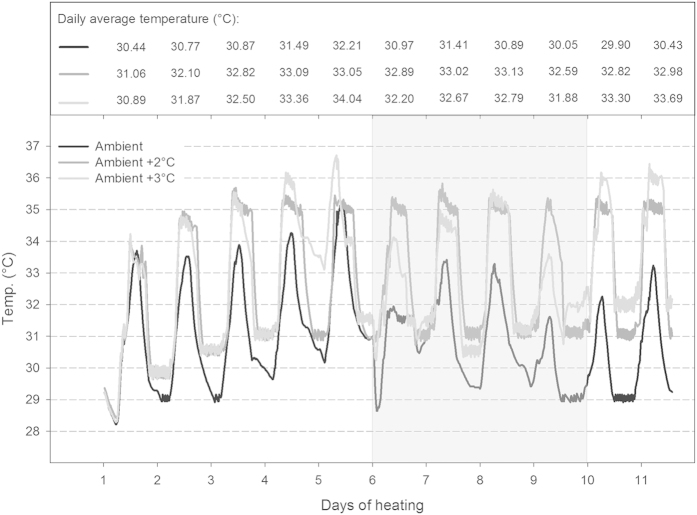
Temperature profiles and daily average temperature (°C) for each treatment over the course of the bleaching experiment. Temperature was gradually increased by 0.6 °C per day over the first 3–5 days until the target temperature in the heat stress treatments was achieved. The shaded area indicates days with unusual weather conditions due to storms, high cloud cover and strong winds. Please note that average day and average night temperatures for each treatment are given in Table 1.

**Figure 3:**
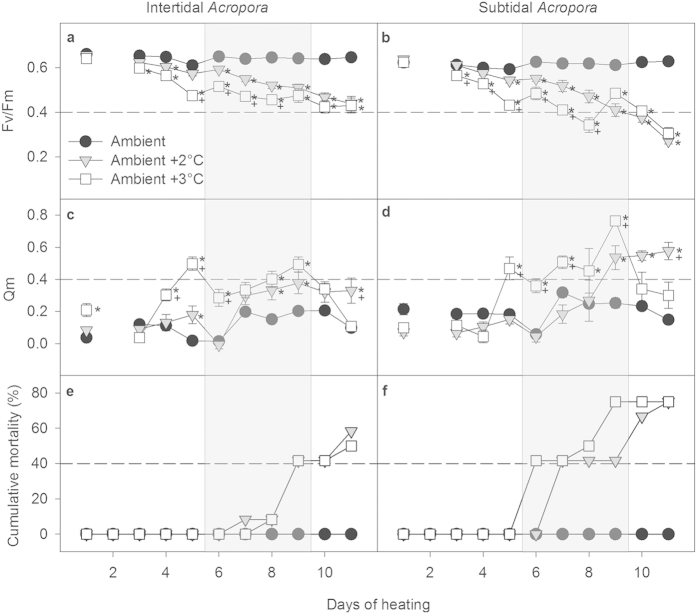
Photochemical efficiency (Fv/Fm) (a,b), excitation pressure over photosystem II (Qm) (c,d) and cumulative mortality (e,f) of intertidal and subtidal *Acropora aspera*. Mean ± SE are shown for (**a**–**d**). Asterisks indicate a significant difference from the ambient control treatment, whereas + indicates a significant difference between ambient +2 and +3 °C treatments. The dashed reference lines were added to highlight differences between intertidal and subtidal corals. The shaded area indicates days with unusual weather conditions due to storms, high cloud cover and strong winds.

**Figure 4:**
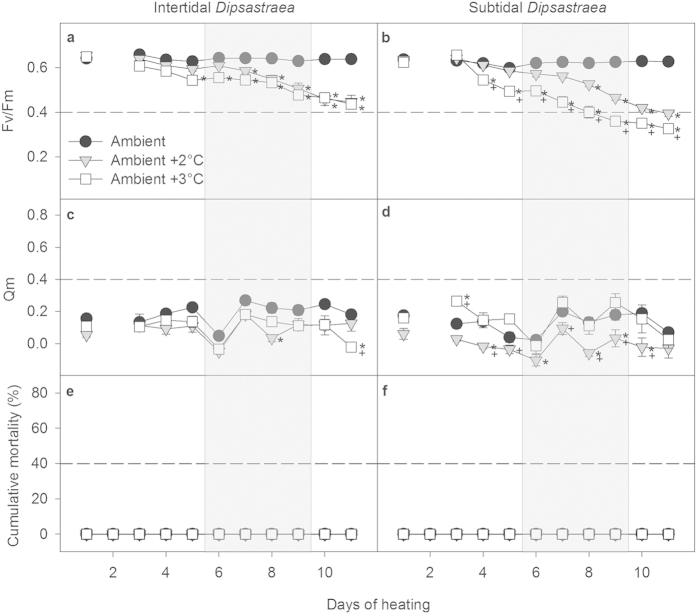
Photochemical efficiency (Fv/Fm) (a,b), excitation pressure over photosystem II (Qm) (c,d) and cumulative mortality (e,f) of intertidal and subtidal *Dipsastraea* sp. Mean ± SE are shown for (**a**–**d**). Asterisks indicate a significant difference from the ambient control treatment, whereas + indicates a significant difference between ambient +2 and +3 °C treatments. The dashed reference lines were added to highlight differences between intertidal and subtidal corals. The shaded area indicates days with unusual weather conditions due to storms, high cloud cover and strong winds.

Our results show that common reef-building corals of the Kimberley can tolerate temperature regimes at which corals from more typical tropical reef environments severely bleach and die, yet nevertheless remain highly susceptible to the stress imposed by daily average temperatures exceeding their maximum monthly mean (MMM) of ~31 °C by 1 °C for only a few days (Figs 2, 3, 4). This finding is consistent with observations from other naturally extreme temperature environment such as the back-reef environment of Ofu Island in American Samoa where heat stress experiments performed on *Acropora* corals showed that temperatures of only 2 °C above the regional MMM caused substantial mortality (up to ~50%) after six days of exposure equivalent to only 11 degree heating days^15^. Similar levels of mortality occurred in subtidal Kimberley *Acropora* corals after exposure to comparable heat stress (Fig. 3F). Similarly, coral reefs in the Gulf experienced a series of natural bleaching events between 1996 and 2011 during which temperatures were elevated ~2 °C above MMM for several weeks resulting in wide-spread mass mortality of *Acropora* spp., severe reductions in coral cover and shifts in coral community composition^35^,^36^,^37^. Collectively, these results suggest that corals already tolerant of naturally higher and more variable temperature environments are nonetheless living precariously close to their physiological limits for enduring thermal stress and that the upper thresholds for coral bleaching and survival are remarkably consistent at 1–3 °C above regional MMM, regardless of location^36^.

It is difficult to establish a single, well-defined temperature as the bleaching threshold for the Kimberley given the highly fluctuating thermal environment and the significant daily temperature variation in our experiment as well as the gradual changes in various physiological metrics that occurred at different times over the course of the study. Clearly, these coral can tolerate average daytime and nighttime temperatures of ~32 °C and ~30 °C for many days (Table 1), respectively, without suffering chronic photoinhibition as indicated by high and largely stable photochemical efficiency (Fv/Fm) in the controls over the course of the experiment (Figs 3 and 4A,B). This was the case for both corals from both environments. It is important to point out, however, that on the one hand maximum light levels in the experiment were lower than those typically encountered by these corals in their native environments^27^, yet on the other hand aerial exposure and stagnant water flow were not simulated in the experiment.

The small drop in Fv/Fm in the control corals on day 5 probably occurred due to ambient peak water temperatures reaching up to 35 °C for short time periods and average daily temperatures exceeding 31 °C for two consecutive days (Fig. 2). This suggests that chronic photoinhibition and thus the earliest onset of bleaching may occur as soon as daily average temperatures exceed the MMM by 1 °C for only a couple of days. However, these findings contrast the lack of any reports of significant coral bleaching for the Kimberley region, despite corals experiencing daily average temperatures of >31 °C on a regular basis in summer (e.g. for 38 d at our study site^27^). This raises the question whether the Kimberley has perhaps warmed significantly over the past decades.

Given the high sensitivity observed in our experiment, it was not surprising that exposure to average daily temperatures of 32-33 °C in the ambient +2 °C treatment (1-2 °C above the MMM) resulted in chronic photoinhibition (Figs 3 and 4A,B) and visible paling for some corals within just 3 and 5 days, respectively. Further, substantial mortality (50–75%) occurred in *Acropora* corals after just 11 days of exposure to average daily temperatures of 32–34 °C (Fig. 3E,F), highlighting that mortality thresholds in this genus are often extremely close to their bleaching thresholds. Overall, our best estimate of a bleaching threshold based on the highly variable temperature treatments and the specific light and flow conditions in this experiment is ~32 °C (daily average temperature, exposure of several days), consistent with NOAA’s approach of defining bleaching thresholds as MMM temperatures +1 °C.

In comparison with other reef environments, this bleaching threshold estimate for the Kimberley is higher than in more traditional coral reef systems but lower than in other naturally extreme temperature environments. For example, bleaching thresholds for many reefs dominated by *Acropora* coral communities across the Great Barrier Reef range from 29–31 °C but are substantially lower when exposure over multiple days is considered^38^. The highest bleaching thresholds reported for reef environments to date come from the Gulf and are 2-3 °C higher than what we have estimated for the Kimberley (34–36 °C vs. ~32 °C)^9^,^35^,^36^. However, summer temperatures in the Gulf are several degrees higher than in the Kimberley with corals spending 4-5 months every year at daily mean temperatures of >30 °C and about 2 months at >33 °C^9^. Combined with the extreme seasonal variation of up to 20 °C^9^, this seems to underlie the extremely high thermal tolerance of Gulf corals. Unfortunately, as yet there is not enough data on the physiological changes these corals undergo under normal and bleaching conditions with which to compare our own results or those from American Samoa.

### The role of the thermal environment in determining bleaching resistance

**Figure 5:**
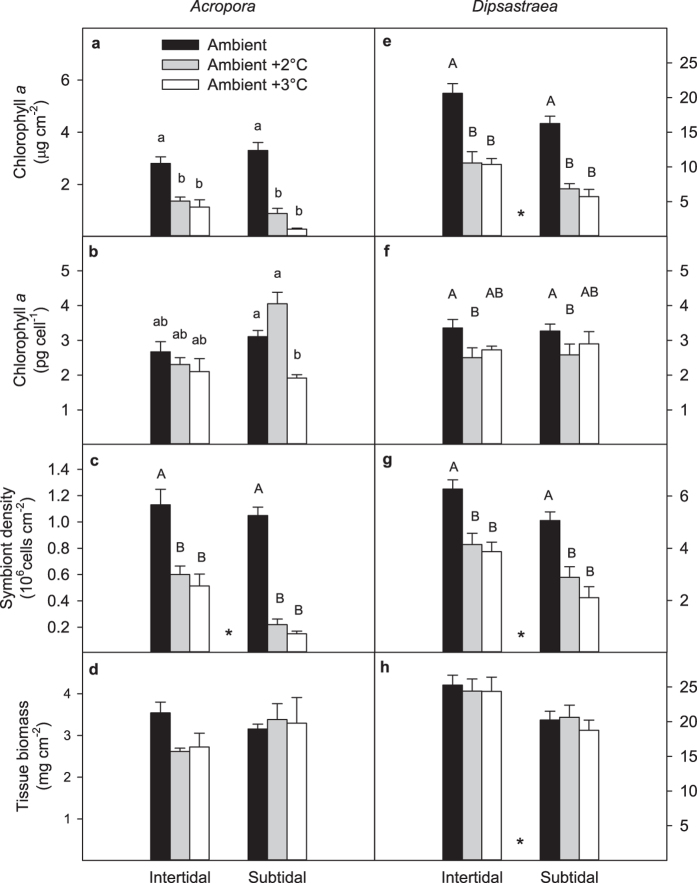
Chlorophyll *a* normalized to (a,e) surface area and (b,f) symbiont cells, symbiont density (c,g) and tissue biomass (d,h) of intertidal and subtidal *Acropora aspera* and *Dipsastraea* sp. after 11 experimental days. Mean ± SE are shown. Asterisks indicate significant effects of environment, whereas upper case letters indicate significant temperature effects. Lower case letters indicate results from Tukey-adjusted multiple pairwise comparisons when there was a significant interaction between environment and temperature. Statistical results in Table S4. Note the different scales for the two corals except in panels (**b**,**f**).

Intertidal corals of both species generally showed higher bleaching resistance and symbiont health than subtidal corals as demonstrated by more modest declines in photochemical efficiency (Fv/Fm), pigment concentrations and symbiont densities as well as lower excitation pressure over photosystem II (Qm), at least for *Acropora* (Figs 3, 4, 5). More importantly, the survival rate of intertidal *Acropora* was higher than that of subtidal *Acropora* under the same levels of heat stress.

Even before the onset of tissue necrosis and death, the bleaching mechanism in *Acropora* differed significantly according to which environment the parent colonies originated from. Heat-stressed intertidal *Acropora* bleached predominantly through the loss of *Symbiodinium* cells whereas subtidal *Acropora* were able to partially compensate for the greater loss of *Symbiodinium* through increased concentrations of chlorophyll *a* in the remaining symbionts (Fig. 5A–C). Overall, the decline in both symbiont cells and chlorophyll *a* per cell in subtidal *Acropora* in the ambient +3 °C treatment indicates that they experienced greater photodamage than those in the ambient +2 °C treatment (Fig. 5B,C). These results are further consistent with higher values of Qm in the +3 °C versus +2 °C treatment (Fig. 3D). In contrast, both intertidal and subtidal heat-stressed *Dipsastraea* predominantly bleached by losing *Symbiodinium* cells rather than chlorophyll *a* per cell (Fig. 5E–G). Such species- and habitat-specific differences in the bleaching mechanism are consistent with other studies^3^,^39^.

Surprisingly, heat-stressed intertidal *Acropora* showed a trend of up to 26% lower tissue biomass than the controls whereas heat-stressed subtidal *Acropora* were able to maintain their tissue biomass (Fig. 5D). This may indicate that the superior ability of intertidal *Acropora* to cope with heat stress comes from an ability to access stored energy reserves such as lipid and protein. These energy reserve pools make up a significant portion of coral tissue biomass^40^ and can play an important role in promoting bleaching resistance and recovery^23^. In *Dipsastraea*, higher overall levels of tissue biomass in intertidal versus subtidal corals could therefore have contributed to their increased bleaching resistance (Fig. 5H). We expect, however, that depletion of energy reserves would be even greater in both *Dipsastraea* and *Acropora* under the more prolonged periods of heat stress that normally precede major natural bleaching events (weeks to months).

The increased thermal tolerance of intertidal versus subtidal corals to heat stress in the present study is consistent with reports showing that corals from back-reef environments are more resistant to thermal stress than corals from the fore reef^12^,^13^,^14^,^15^, although not in all cases^16^. Since back-reef environments typically experience much larger fluctuations and extremes in temperature and other parameters, this is consistent with our findings of increased thermal tolerance for intertidal compared to subtidal corals because the intertidal environment represents much more extreme temperature conditions than the subtidal^27^ (Fig. 1, see Methods). However, it is likely that the higher light intensities and UV levels experienced by corals in the intertidal act in concert with the more extreme temperature fluctuations to increase heat tolerance as some corals can acquire resistance to heat-induced bleaching via prior exposure to high solar radiation^41^. Similarly, other environmental factors such as pH and oxygen can also vary significantly in tidal environments (though pH fluctuations are moderate at our study site^27^) and may affect coral thermal tolerance.

Importantly, the genetic type of *Symbiodinium* did not differ between environments and temperature treatments. Thus, this study confirms that more extreme fluctuations in temperature enhance bleaching resistance even without undergoing substantial changes to the symbiont genotype. However, it is less clear whether this enhancement in thermal stress resistance is the result of acclimatisation, natural selection and/or adaptation of the coral holobiont given that the two environments are within <500 m and the intertidal pool is well-flushed during high tides. Nonetheless, genetically distinct coral host populations can exist between lagoon and reef slope environments^42^, and even in the absence of genetic population substructures, genetic differences can still provide a mechanism for increased heat tolerance^43^,^44^. Further genetic studies are needed to determine whether the same is true across intertidal and subtidal habitats in the Kimberley region.

### Other factors determining thermal tolerance

The genetic type of *Symbiodinium* spp. can play a significant role in determining thermal tolerance because some types (in particular *S. trenchii*^45^ within clade D) have been found to perform better at high temperatures than others^21^,^46^. Further, there is evidence that more extreme temperature environments often support higher abundance of corals hosting clade D^21^. It may therefore be surprising that the thermally tolerant Kimberley corals in this study all hosted clade C (chloroplast 23S type Cp1 with the exception of one subtidal *Acropora* colony that hosted Cp20); however, prior studies have also found that symbionts in *Acropora* corals from the Kimberley are dominated by clade C^47^ and that *Acropora* in Western Australia generally has a high symbiont specificity for clade C across a large latitudinal range^47^,^48^. We know of no other studies that have analysed symbiont type in Western Australian *Dipsastraea* corals, but Pacific congeners are also typically dominated by clade C^49^.

Although these findings are certainly interesting, it is important to caution that inferring thermal tolerance from cladal resolution can be misleading. For example, it has been shown that clade D comprises several species that are physiologically and ecologically distinct^45^ and that significant functional diversity also exists within clade C^50^-though this was not assessed in our study. This and other studies are therefore making it increasingly clear that resistance to heat stress can be achieved without the presence of clade D. For example, *Symbiodinium* C3 dominates corals in the southern Gulf, one of the hottest environments in the world supporting coral growth^11^,^51^, although it was recently shown that this particular C3 variant from the Gulf represents a new thermotolerant species (*S. thermophilum*)^51^. Similarly, *Porites lobata* in American Samoa hosted C15 independent of whether they grew on the fore reef or the warmer and more variable back reef^42^. Further, *Symbiodinium* C1 can be adapted locally to high temperatures^52^ and increased resistance to thermal stress can be achieved without changes in symbiont type due to acclimation of the coral holobiont^12^,^53^. It is therefore likely that both *Symbiodinium* and the coral host are locally adapted to the high temperature environment of the Kimberley, and that this is enhanced by the extreme temperature fluctuations of the intertidal environment (Fig. 1).

Another important factor determining thermal tolerance is coral morphology. Branching *Acropora* in this study was much more susceptible to coral bleaching and mortality than massive *Dipsastraea*, consistent with well-established patterns of morphologically dependent bleaching susceptibility^18^,^19^, which are hypothesised to result from differences in tissue thickness. Typically, massive corals have thicker tissues than branching corals, which is consistent with more than 5 times higher tissue biomass per area in *Dipsastraea* compared to *Acropora* (Fig. 5D,H). Thicker tissues can provide increased protection from light, more efficient self-shading of the symbiont cells and higher levels of energy reserves, thus improving overall resistance to light and heat stress^18^,^22^.

### Implications for the future of Kimberley coral reefs

In contrast to the wide-spread use of constant temperatures in bleaching experiments, all treatments in the present study experienced significant daily temperature variation (up to 5 °C), thus mimicking the *in situ* conditions experienced by these corals. This is also important because lower night temperatures can significantly reduce bleaching and mortality during periods of thermal stress^54^. High flow rates, which are characteristic for Kimberley coral reefs, can further help reduce mortality and photoinhibition during thermal stress^55^ and are therefore critical to properly assess bleaching susceptibility in a given reef habitat. The use of mini-flumes in this study provided experimental corals with moderate flow (12–15 cm s^−1^), which likely helped to moderate the amount of thermal stress received. However, we did not simulate aerial exposure and stagnant flow which would likely have further augmented heat and photooxidative stress during low tide slack water periods. It is therefore possible that during natural bleaching events in this region, bleaching susceptibility and mortality may be even higher than observed in this experiment, particularly in the intertidal and shallow subtidal when coinciding with mid-day spring tides. On the other hand, the high turbidity of Kimberley waters could also potentially mitigate light and heat stress to some extent and it remains to be determined how these factors play out during natural bleaching events.

Overall, our findings and those from previous work^12^,^15^,^23^,^43^,^56^,^57^ clearly show that corals exhibit significant potential for acclimatization and/or adaptation and that the thermal (micro)environment plays a key role in this process. Specifically, highly variable temperatures rather than just high mean temperatures alone appear to enhance the tolerance of coral to thermal stress. Such adaptive processes have important implications for predicting the spatial and temporal patterns of future coral bleaching events and may significantly delay the onset of frequent severe bleaching events worldwide^6^.

## Methods

### Collection Sites and Thermal Environment

Coral fragments of branching *Acropora aspera* (~5 cm) and massive *Dipsastraea* sp. (formerly *Favia*^31^, 3-4 cm diameter) were collected in April 2014 from Shenton Bluff, Cygnet Bay, Kimberley region, Western Australia. They were collected from shallow depth (<2 m) in two different thermal environments, the intertidal and subtidal, which are described in detail elsewhere^27^. The intertidal environment (16°28′45.8″ S, 123°2′41.3″ E; referred to as “isolated” in ref. 27) is a small shallow pool (ca. 200 × 100 m) that becomes isolated from the surrounding waters of King Sound during low tides. The associated slack water period lasts for up to 4 hours and the shallower corals become exposed to air during this time while the submerged corals are subject to stagnant flow condition. Coral colonies were collected throughout the intertidal, thus representing both genotypes that get exposed to air regularly as well as genotypes that remain largely submerged during low tides. Temperature logger data from 2011–2013 showed that the daily variation in seawater temperatures in this pool is up to 7 °C, while the seasonal range is 22 °C to 31.5 °C based on a 7-day moving average of daily mean temperatures^27^ (Fig. 1). Maximum monthly mean (MMM) temperatures of 30.9 °C and 31.2 °C were recorded in December 2011 and February 2013, respectively^27^.

In contrast, the subtidal environment (16°28′46.8″ S, 123°2′36.6″ E; referred to as “subtidal” in ref. 27) represents a more moderate thermal environment that experiences only up to 3 °C daily temperature variation although the seasonal temperature range in the subtidal is the same as in the intertidal^27^ (22 °C to 31.5 °C; Fig. 1). Similarly, MMM temperatures were 31.1, 30.8 and 31.3 °C in December 2010, December 2011 and February 2013, respectively^27^. Corals in this environment are typically not exposed to air during low tides, except during the most extreme spring low tides (i.e., only a few days per year). Although pH fluctuations are larger in the intertidal than the subtidal, they generally do not exceed ~0.1 units^27^.

Colonies (n = 12 for *Acropora*, n = 10 for *Dipsastraea*) were selected at least 10 m apart to increase the probability that different genotypes of the same species were selected. For *Acropora*, fragments were taken from the top part of large colonies, whereas *Dipsastraea* fragments were collected from medium sized colonies up to 25 cm in diameter. Four fragments were collected from each parent colony per environment and species, one for each of the three temperature treatments and a fourth fragment stored in 100% ethanol to determine the *Symbiodinium* type of each parent colony (see below). Coral fragments were then glued onto plastic tiles and maintained in shaded outdoor, flow-through seawater tanks (see below). Corals were allowed to acclimate at ambient seawater temperature (day: ~32 °C, night: ~30 °C) for 1 week prior to the start of the experiment. During that time, they were stained with alizarin red at a concentration of ~5 mg/L for 9 hours during daylight.

### Coral Bleaching Experiment

The bleaching experiment was conducted from 25 April to 5 May 2014 (11 days) at the Kimberley Marine Research Station located at Cygnet Bay Pearl Farm. Since seasonal variation in bleaching thresholds can occur^16^, we wanted to test the thermal resilience of the Kimberley coral at the end of the summer when temperature stress is most likely to occur. Bleaching thresholds are as yet unknown for coastal Kimberley regions and were therefore estimated to be >32 °C based on MMM data from temperature loggers deployed in previous years.

Coral fragments were randomly assigned to each of three temperature treatments: (1) ambient control (average day: 31.9 °C, average night: 29.9 °C – see Table 1), (2) ambient +2 °C and (3) ambient +3 °C. Each temperature treatment consisted of two separate 43 L flow-through tanks (one tank each for the corals from the inter- and subtidal environment, respectively) fed from one 140 L sump which received flow-through ocean water. Thus, there were a total of three sumps and six flow-through tanks (2 species × 3 treatments = 6 tanks). Temperature in the sump was controlled using titanium heaters (Wei Pro, 1000 W) connected to a temperature controller (Auber Instr. TD100A).Temperature was gradually increased by 0.6 °C per day until the target temperature was achieved to prevent heat shock. Importantly, a maximum daily temperature variation of 4-5 °C was maintained in all treatments (Fig. 2) to better mimic the naturally variable thermal conditions. HOBO temperature loggers recorded seawater temperature every 15 min in all six tanks.

Since water flow can significantly affect thermal tolerance^55^, tanks were designed as miniflumes (length 117 cm, width 25 cm, height 29 cm; water depth 15 cm) to allow for more realistic flow conditions. Two submersible pumps (Macro Aqua) per tank generated flow rates of 12–15 cm s^−1^ (determined from the timed passage of dye). Seawater renewal rate was 3 L min^−1^ for each treatment, resulting in a turnover time of ~15 minutes per treatment. Incoming seawater was filtered to a nominal size of 10 μm so although corals were not fed during the experiment, they nonethless had access to some natural particulate (<10 μm) and dissolved organic matter as well as dissolved inorganic nutrients provided by the incoming seawater. Shade cloth reduced incoming maximum photosynthetically active radiation (PAR) levels to 500 and 400 μmol m^−2^ s^−1^ just below the water surface and at the bottom of the tanks, respectively (measured using an Apogee MQ-200 cosine-corrected planar PAR-meter). These light levels were considerably lower than those in the natural environment^27^ to avoid undue light stress in the subtidal corals.

Although we had planned on conducting the experiment for several weeks, the experiment was ended after 11 days due to significant mortality in some treatments (see Results). After the experiment was terminated, all corals were frozen, transported back to the University of Western Australia (UWA) under liquid nitrogen and stored at −80 °C until further analysis.

### Monitoring and Characterisation of Treatment Conditions

Seawater temperature, salinity and conductivity were measured daily in all six tanks (two per treatment) using a YSI 85 multi-sensor. Seawater samples for total alkalinity (TA) and nutrient samples were taken from each of the six tanks every three days. pH was measured in each tank within 15 min of collecting the water samples using a Schott handylab pH 12 pH meter. Water samples were filtered using glass fibre filters with 0.7 μm nominal pore size (Whatman GF/F), collected in screw-top Nalgene HDPE bottles and stored frozen until analysis. TA was determined by titration from a spectrophotometrically determined end-point pH^58^. Treatment xCO_2_ (dry air), aragonite saturation state (Ω_arag_), and pH_T_ were calculated using the program CO2SYS^59^ based on measured pH and alkalinity. An aliquot of the water samples collected for TA analysis was used to measure concentrations of ammonium (NH_4_^+^, ± 0.2 μM), nitrate (NO_3_^−^, ± 0.05 μM) and phosphate (HPO_4_^2−^, ±0.02 μM) using a QuikChem 8500 Series 2 Flow Injection Analysis (FIA) System (Lachat Instrument, USA) according to standard colorimetric methods as provided by the manufacturer.

In addition to calculating average day and night water temperatures for each temperature treatment, degree heating days (DHD) and heating rate (HR) were calculated^60^. Although these indicators of thermal stress are not typically used in an experimental context, we decided to use them here as they provide a measure of cumulative thermal stress and are thus more useful in characterising the experimental heating treatments and facilitating comparison with *in situ* bleaching events. Since long-term mean summer temperatures (LMST) are not available for the Kimberley region and experimental control temperatures are more relevant in an experimental context, DHD were calculated as follows:


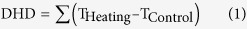


where T_Heating_ is the average daily temperature in the respective heating treatment and T_Control_ is the average daily temperature in the control treatment over the course of the bleaching experiment. To account for the rate of temperature increase, HR was also calculated as follows:


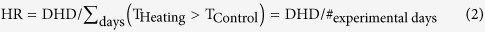


### Physiological and Genetic Analyses

#### Mortality

Coral mortality was visually assessed for each fragment daily in the morning and during fluorescence measurements at noon.

#### Endosymbiont type

Initial algal endosymbiont types were determined from small (1-2 cm) biopsies, which were removed from all parent colonies sampled during coral collection and stored in 100% ethanol. To detect any changes in symbiont type occurring during the experiment, biopsies were removed from each surviving coral fragment at the end of the experiment and stored in 100% ethanol. *Symbiodinium* in five samples per treatment and species collected at the start and end of the experiment (unless less than five fragments per treatment survived) were genotyped. The same coral colonies were analysed for all intertidal and subtidal temperature treatments, respectively.

Total DNA was extracted using the DNeasy Blood and Tissue Kit (Qiagen) following the manufacturer’s instructions with an initial overnight incubation at 56 °C. *Symbiodinium* chloroplast 23S rDNA domain V was amplified in PCR using forward 23S1 (5´ GGC TGT AAC TAT AAC GGT CC 3´) and reverse 23S2 (5´ CCA TCG TAT TGA ACC CAG C 3´) primers^61^. PCR reactions contained 0.5U JumpStart^TM^ Taq DNA polymerase (Sigma-Aldrich), 1 X PCR buffer, 1 mM MgCl_2_, 20 μg BSA, 0.2 mM each dNTP, 0.2 μM each primer, and 1 μl DNA template made up to a 30 μl volume with sterile deionized water. PCR was performed in an Eppendorf Mastercycler® with 5 min at 94 °C followed by 35 cycles of 30  s at 95 °C, 30 s at 55 °C, and 1 min at 72 °C, and ended with a final 10 min extension at 72 °C. 23S rDNA amplicons were purified and sequenced in both directions at the Australian Genome Research Facility (Perth node). Chromatograms were inspected and edited in Geneious 6.1.6. Chloroplast 23S rDNA haplotypes were identified by performing a nucleotide BLAST search in NCBI.

#### Photophysiology

Effective quantum yield (ΔF/Fm′) of chlorophyll *a* fluorescence in each coral fragment was measured daily at noon (except for day 2) to assess the photochemical efficiency of coral in the light-adapted state. Maximum quantum yield (Fv/Fm) of chlorophyll *a* fluorescence in each coral fragment was also measured daily 1 hour after sunset (except for day 2) to assess the photochemical efficiency in the dark-adapted state. All photochemical measurements were made using a diving-PAM underwater fluorometer (Walz, Germany) with the following settings: measuring light intensity = 3, saturation pulse intensity = 12, saturation pulse width = 0.8 s, gain = 6 and 5 for *Acropora* and *Dipsastraea*, respectively, and damping = 2. Measurements were made at a constant distance of 3 mm from the coral tissue, approx. 1 cm below the tip or growing edge. Due to the fixed position and orientation of corals within tanks, a similar part of the coral was measured at each time point. The maximum excitation pressure over photosystem II (Qm)^62^, which is an indicator of symbiont performance at peak sunlight, was calculated as Qm = 1−(ΔF/Fm’)/(Fv/Fm), with values close to 1 indicating photoinhibition and values close to 0 indicating light-limitation of photosynthesis under maximum irradiance.

#### Tissue biomass, chlorophyll a and symbiont density

Coral tissue was removed from the skeleton using either an airbrush (*Acropora*) or a waterpik (*Dipsastraea*). A 3–6 ml aliquot of the resulting tissue slurry was then dried at 60 °C in pre-combusted aluminium pans to constant weight and ashed in a muffle furnace at 500 °C for 4 hours^63^. Ash-free dry weight (=tissue biomass) was determined as the difference between dry and ash weight and standardized to surface area, which was estimated using the simple geometry technique for *Acropora* and the aluminium foil technique^64^ for *Dipsastraea*. The remaining tissue slurry was separated into animal host and symbiont fraction via centrifugation. Chlorophyll *a* and *c*_*2*_ were extracted in 100% acetone in the dark at 4 °C for 24 hours, determined spectrophotometrically using the equations of Jeffrey and Humphrey^65^ and standardized to both surface area and cell density. Symbiont cell density was calculated using 8 replicate counts on an improved Neubauer hemocytometer and standardized to surface area.

### Statistical Analyses

Non-parametric one-way analysis of variance (ANOVA) was used to test for significant differences in tank conditions (i.e., average day and night temperature, T_Heating_ − T_Control_, pH_T_, pCO_2_, total alkalinity, saturation state and nutrient concentrations) between intertidal and subtidal tanks within each temperature treatment.

For Fv/Fm and Qm, generalized linear mixed model (GLMM) analysis was used to test for the effect of time (=days of heating), temperature, and environment for each species individually. Time was fixed with ten levels (days 1, 3–11 – no measurements were performed on day 2), temperature was fixed with three levels (ambient, ambient +2 °C, ambient +3 °C) and environment was fixed with two levels (intertidal, subtidal). Parent colony was a random factor nested within environment. For chlorophyll *a* and *c*_*2*_ (per area and per cell), endosymbiont density and tissue biomass, GLMM analysis was used to test for the effects of temperature, environment and parent colony for each species individually. Tukey adjusted *p*-values were used for post hoc tests when main effects were significant. When a significant interaction was observed, multiple pair-wise comparisons were conducted using Tukey adjusted *p*-values.

Since all fragments were exposed to identical conditions except temperature during the bleaching treatments, any differences in the observed responses were due to temperature and environment effects alone and independent of seasonal variation. *P*-values ≤0.05 were considered significant. Statistical analyses were performed using SAS software version 9.3.

## Additional Information

**How to cite this article**: Schoepf, V. *et al*. Limits to the thermal tolerance of corals adapted to a highly fluctuating, naturally extreme temperature environment. *Sci. Rep*. **5**, 17639; doi: 10.1038/srep17639 (2015).

## Supplementary Material

Supplementary Information

## Figures and Tables

**Table 1:** Average conditions for each of the six tanks maintained at three different temperature regimes.

	Ambient		Ambient +2 °C		Ambient +3 °C	
	Intertidal	Subtidal	Intertidal	Subtidal	Intertidal	Subtidal
**Day Temp. (°C)**	31.86 ± 0.27	31.89 ± 0.26	34.15 ± 0.16	34.37 ± 0.18	33.89 ± 0.28	34.05 ± 0.30
**Night Temp. (°C)**	29.84 ± 0.21	29.85 ± 0.19	31.22 ± 0.24	31.01 ± 0.20	31.38 ± 0.33	31.31 ± 0.31
**Degree Heat. Days**	0	0	20.41	20.30	19.85	20.08
**Heating Rate**	0	0	1.86	1.85	1.80	1.83
**pH**_**T**_	8.03 ± 0.03	8.03 ± 0.03	8.00 ± 0.02	8.00 ± 0.02	7.99 ± 0.01	7.98 ± 0.01
**pCO**_**2**_ **(μatm)**	395.3 ± 34.67	396.3 ± 33.51	423.3 ± 21.29	432.2 ± 22.15	445.9 ± 14.49	454.6 ± 12.21
**TA (μmol kg**^**−1**^)	2215 ± 8.89	2218 ± 5.56	2224 ± 6.92	2224 ± 4.92	2226 ± 6.73	2224 ± 6.14
**Ω**_**arag**_	3.8 ± 0.14	3.9 ± 0.13	3.9 ± 0.15	3.9 ± 0.14	3.8 ± 0.12	3.8 ± 0.10
**NH**_**4**_^**+**^**(μmol N L**^**−1**^)	0.92 ± 0.05	0.90 ± 0.05	0.85 ± 0.06	0.87 ± 0.10	0.87 ± 0.01	0.92 ± 0.02
**NO**_**3**_^**−**^ **(μmol N L**^**−1**^)	0.33 ± 0.02	0.30 ± 0.02	0.28 ± 0.01	0.28 ± 0.01	0.30 ± 0.01	0.35 ± 0.05
**PO**_**4**_^**3−**^ **(μmol P L**^**−1**^)	0.06 ± 0.00	0.06 ± 0.01	0.04 ± 0.00	0.04 ± 0.02	0.04 ± 0.02	0.05 ± 0.03

Mean ± 1 SE are shown. Temperature ( = Temp.) data are based on daily averages obtained from logger data (n = 11), whereas carbonate chemistry and nutrient data are based on biweekly measurements (n = 3). Heat. = Heating, TA = total alkalinity, Ω_arag_ = saturation state for aragonite, NH_4_^+^ = ammonium, NO_3_^−^ = nitrate, PO_4_^3−^ = phosphate.
