# Supplementary material for: Limits to the thermal tolerance of corals adapted to a highly fluctuating, naturally extreme temperature environment
Source: Sci Rep. 2015 Dec 2;5:17639. doi: 10.1038/srep17639 (PMC4667274; doi:10.1038/srep17639)
Supplement: Supplementary Information [file srep17639-s1.pdf]

Limits to the thermal tolerance of corals adapted to a highly fluctuating, naturally extreme temperature environment

Verena Schoepf[1,2,3,*], Michael Stat[4], James L. Falter[1,2,3] and Malcolm T. McCulloch[1,2,3]

[1] School of Earth and Environment and ARC Centre of Excellence for Coral Reef Studies, The University of Western Australia, Crawley, WA, Australia

[2] UWA Oceans Institute, The University of Western Australia, Crawley, WA, Australia

[3] The Western Australian Marine Science Institution, Perth, WA, Australia

[4] Trace and Environmental DNA (TrEnD) Laboratory, Department of Environment and Agriculture, Curtin University, Perth, WA, Australia

* Corresponding author. Email: schoepf.4@osu.edu

Supplemental Materials

Supplemental Table S1. Results from one-way ANOVAs to test for significant differences in tank conditions between intertidal and subtidal tanks within each temperature (temp.) treatment. df=degrees of freedom, SS=sum of squares, TA = total alkalinity, Ω_{arag} = saturation state for aragonite.

**Variable**	**Temp.**	**df**	**SS**	***F*-statistic**	***P*-value**
Day Temp.	Ambient	1	0.0036	0.0046	0.9467
	Ambient +2°C	1	0.2728	0.8392	0.3705
	Ambient +3°C	1	0.1473	0.1592	0.6941
Night Temp.	Ambient	1	0.0006	0.0012	0.9726
	Ambient +2°C	1	0.2426	0.4478	0.5110
	Ambient +3°C	1	0.0277	0.0241	0.8782
$T_{Heating}$ - $T_{Control}$	Ambient	n/a	n/a	n/a	n/a
	Ambient +2°C	1	0.0006	0.0012	0.9728
	Ambient +3°C	1	0.0024	0.0034	0.9541
pH_T	Ambient	1	0.0000	0.0059	0.9424
	Ambient +2°C	1	0.0001	0.0678	0.8074
	Ambient +3°C	1	0.0002	0.5294	0.5072
pCO_2	Ambient	1	1.3067	0.0004	0.9855
	Ambient +2°C	1	118.1041	0.0834	0.7871
	Ambient +3°C	1	113.7962	0.2112	0.6697
TA	Ambient	1	10.1140	0.0613	0.8166
	Ambient +2°C	1	0.2166	0.0020	0.9664
	Ambient +3°C	1	8.8088	0.0708	0.8033
Ω_{arag}	Ambient	1	0.0000	0.0000	1.0000
	Ambient +2°C	1	0.0017	0.0250	0.8820
	Ambient +3°C	1	0.0014	0.0375	0.8559
Ammonium	Ambient	1	0.0012	0.1370	0.7301
	Ambient +2°C	1	0.0005	0.0232	0.8862
	Ambient +3°C	1	0.0039	3.3611	0.1407
Nitrate	Ambient	1	0.0013	1.2535	0.3256
	Ambient +2°C	1	0.0000	0.0628	0.8145
	Ambient +3°C	1	0.0036	1.0627	0.3609
Phosphate	Ambient	1	0.0000	0.0672	0.8082
	Ambient +2°C	1	0.0000	0.0240	0.8843
	Ambient +3°C	1	0.0001	0.0723	0.8014

Supplemental Table S2. Results from two generalized linear mixed model analyses to test for the effects of time, temperature (=Temp.), and environment (=Env.) on Fv/Fm and Qm of *Acropora aspera*. *P*-values ≤0.05 are highlighted in bold. Num df = numerator degrees of freedom, den df = denominator degrees of freedom.

Effect	Num df	Den df	*F*-statistic	*P*-value
**Fv/Fm**				
Temp.	2	44	370.39	**<0.0001**
Time	9	480	189.50	**<0.0001**
Temp. x Time	18	480	63.41	**<0.0001**
Env.	1	22	34.45	**<0.0001**
Temp. x Env.	2	44	5.41	**0.0079**
Env. x Time	9	480	7.80	**<0.0001**
Temp. x Env. x Time	18	480	6.51	**<0.0001**
**Qm**				
Temp.	2	44	22.05	**<0.0001**
Time	9	477	57.17	**<0.0001**
Temp. x Time	18	477	18.96	**<0.0001**
Env.	1	22	3.18	0.0882
Temp. x Env.	2	44	0.33	0.7224
Env. x Time	9	477	4.61	**<0.0001**
Temp. x Env. x Time	18	477	5.45	**<0.0001**

Supplemental Table S3. Results from two generalized linear mixed model analyses to test for the effects of time, temperature (=Temp.), and environment (=Env.) on Fv/Fm and Qm of *Dipsastraea* sp. *P*-values ≤0.05 are highlighted in bold. Num df = numerator degrees of freedom, den df = denominator degrees of freedom.

Effect	Num df	Den df	*F*-statistic	*P*-value
**Fv/Fm**				
Temp.	2	36	45.16	**<0.0001**
Time	9	485	176.94	**<0.0001**
Temp. x Time	18	485	44.36	**<0.0001**
Env.	1	18	5.88	**0.0260**
Temp. x Env.	2	36	3.10	0.0573
Env. x Time	9	485	6.97	**<0.0001**
Temp. x Env. x Time	18	485	4.82	**<0.0001**
**Qm**				
Temp.	2	36	21.23	**<0.0001**
Time	9	485	23.06	**<0.0001**
Temp. x Time	18	485	2.55	**0.0005**
Env.	1	18	1.64	0.2172
Temp. x Env.	2	36	9.00	**0.0007**
Env. x Time	9	485	3.26	**0.0007**
Temp. x Env. x Time	18	485	0.96	0.5091

Supplemental Table S4. Results from generalized linear mixed models to test for the effects of temperature (=Temp.) and environment (=Env.) on chlorophyll *a* per area, chlorophyll *a* per cell, symbiont density (=Dens.) and tissue biomass (=Biom.) of *Acropora aspera* and *Dipsastraea* sp. Post hoc Tukey tests were used when main effects (but no interaction terms) were significant. *P*-values ≤0.05 are highlighted in bold. Num df = numerator degrees of freedom, den df = denominator degrees of freedom. NB=ambient control, BL=ambient +2°C, BH=ambient+3°C. IT=Intertidal, ST=subtidal.

Factor	Effect	Num df	Den df	*F*-statistic	*P*-value	Tukey
Acropora aspera						
Chl *a* area^{-1}	Temp.	2	13	55.22	**<0.0001**	
	Env.	1	22	2.45	0.1317	
	Temp. x Env.	2	13	6.07	**0.0137**	
Chl *a* cell^{-1}	Temp.	2	13	9.26	**0.0032**	
	Env.	1	22	1.10	0.3055	
	Temp. x Env.	2	13	4.60	**0.0308**	
Dens.	Temp.	2	13	44.95	**<0.0001**	NB > BL=BH
	Env.	1	22	4.36	**0.0486**	IT > ST
	Temp. x Env.	2	13	1.23	0.3233	
Biom.	Temp.	2	13	2.40	0.1294	
	Env.	1	22	0.52	0.4778	
	Temp. x Env.	2	13	2.44	0.1264	
***Dipsastraea* sp.**						
Chl *a* area^{-1}	Temp.	2	36	110.72	**<0.0001**	NB > BL=BH
	Env.	1	18	9.64	**0.0061**	IT > ST
	Temp. x Env.	2	36	0.19	0.8297	
Chl *a* cell^{-1}	Temp.	2	36	5.72	**0.0069**	NB=BH > BH=BL
	Env.	1	18	0.04	0.8392	
	Temp. x Env.	2	36	0.17	0.8481	
Dens.	Temp.	2	36	65.42	**<0.0001**	NB > BL=BH
	Env.	1	18	9.17	**0.0072**	IT > ST
	Temp. x Env.	2	36	0.78	0.4667	
Biom.	Temp.	2	36	0.47	0.6275	
	Env.	1	18	7.42	**0.0139**	IT > ST
	Temp. x Env.	2	36	0.26	0.7736	

Supplemental Table S5. Results from generalized linear mixed models to test for the effects of temperature (=Temp.) and environment (=Env.) on chlorophyll c_2 per area and chlorophyll c_2 per cell of *Acropora aspera* and *Dipsastraea* sp. Post hoc Tukey tests were used when main effects (but no interaction terms) were significant. *P*-values ≤0.05 are highlighted in bold. Num df = numerator degrees of freedom, den df = denominator degrees of freedom. NB=ambient control, BL=ambient+2°C, BH=ambient+3°C. IT=Intertidal, ST=subtidal.

Factor	Effect	Num df	Den df	*F*-statistic	*P*-value	Tukey
Acropora aspera						
Chl c_2 area^{-1}	Temp.	2	13	39.22	**<0.0001**	NB > BL=BH
	Env.	1	21	16.27	**0.0006**	IT > ST
	Temp. x Env.	2	13	3.46	0.0625	
Chl c_2 cell^{-1}	Temp.	2	13	10.78	**0.0017**	
	Env.	1	21	2.50	0.1288	
	Temp. x Env.	2	13	6.81	**0.0095**	
***Dipsastraea* sp.**						
Chl c_2 area^{-1}	Temp.	2	35	52.96	**<0.0001**	NB > BL=BH
	Env.	1	18	5.89	**0.0259**	IT > ST
	Temp. x Env.	2	35	1.30	0.2858	
Chl c_2 cell^{-1}	Temp.	2	35	0.78	0.4681	
	Env.	1	18	3.82	0.0664	
	Temp. x Env.	2	35	0.74	0.4850	

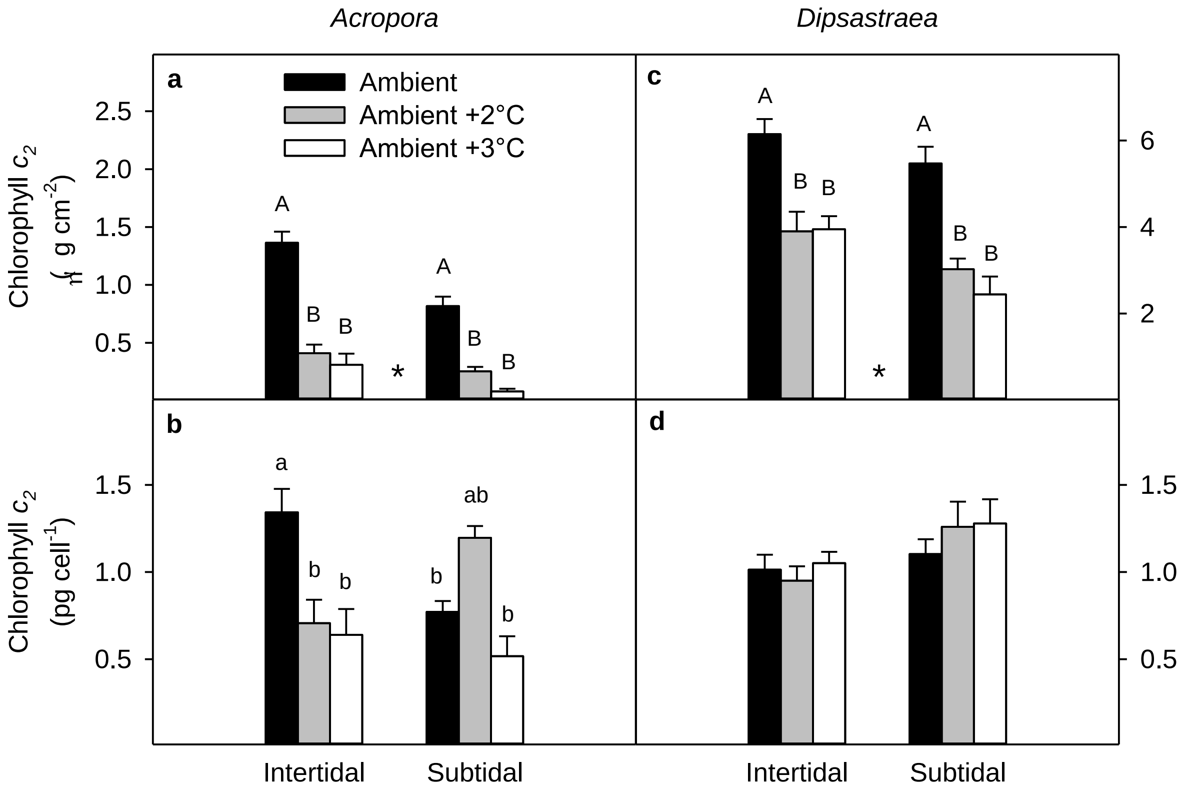

Supplemental Figure S1. Chlorophyll c_2 normalized to (a, c) surface area and (b, d) symbiont cells of intertidal and subtidal *Acropora aspera* and *Dipsastraea* sp. Mean ± SE are shown. Asterisks indicate significant effects of environment, whereas upper case letters indicate significant temperature effects. Lower case letters indicate results from Tukey-adjusted multiple pairwise comparisons when there was a significant interaction between environment and temperature. Statistical results in Table S5. Note the different scales for the two corals in panels a and c.
